# Impact of targeted educational intervention towards public knowledge and perception of antibiotic use and resistance in the state of Perak, Malaysia

**DOI:** 10.1186/s13756-021-00892-0

**Published:** 2021-02-04

**Authors:** Kah Shuen Thong, Chee Tao Chang, Ming Lee, Jason Choong Yin Lee, Hoo Seng Tan, Asrul Akmal Shafie

**Affiliations:** 1 Department of Pharmacy, Raja Permaisuri Bainun Hospital, Ministry of Health Malaysia, Ipoh, Malaysia; 2 Clinical Research Centre, Raja Permaisuri Bainun Hospital, Ministry of Health Malaysia, Ipoh, Malaysia; 3 Department of Pharmacy, Klinik Kesihatan Kampung Simee, Ministry of Health Malaysia, Ipoh, Malaysia; 4 Perak Pharmaceutical Services Division, Ministry of Health Malaysia, Ipoh, Malaysia; 5 School of Pharmaceutical Sciences, Universiti Sains Malaysia, Gelugor, Malaysia

**Keywords:** Health education, Knowledge, Perception, Antibiotic,, Antibiotic resistance, Malaysia

## Abstract

**Background:**

Antibiotic resistance is a major public health concern, accelerated by antibiotic overuse. Inadequate knowledge among the public has been associated with inappropriate use of antibiotics. This study determined the impact of a self-developed educational leaflet for addressing specific knowledge gaps in antibiotic use among the public.

**Methods:**

This was an experimental study conducted at five hospitals and 20 primary health care clinics in the state of Perak. Adults over 18 years of age were recruited using sequential sampling. The first phase of data collection consisted of a pre-intervention assessment, an educational session, and an immediate post-intervention assessment. Each educational session was conducted by trained pharmacists and lasted approximately 15 min for each participant. A two-week post-intervention assessment was then conducted via a phone call to re-assess the participants using the same questionnaire.

**Results:**

Out of 300 questionnaires distributed, 234 were completed for our study. The mean age of participants was 40.7 ± 14.6 years old. Most of the respondents were female (143, 61.1%), Malay (162, 69.2%), and had tertiary education (162, 69.2%). A mean score was generated for each domain, with knowledge towards antibiotic resistance: 2.83 ± 1.28 pre-intervention, 3.76 ± 0.62 immediate post-intervention, and 3.67 ± 0.78 two-weeks post-intervention (total score: 4.00); knowledge towards antibiotic use: 2.03 ± 1.56 pre-intervention, 4.56 ± 1.46 immediate post-intervention, and 4.32 ± 1.48 two-weeks post-intervention (total score: 6.00); perception towards antibiotic use: 2.83 ± 1.38 pre-intervention, 4.25 ± 1.06 immediate post-intervention, and 4.22 ± 1.02 two-weeks post-intervention (total score: 5.00). Significant improvement in the mean scores were found before and after intervention in all domains (*p* < 0.001).

**Conclusion:**

The educational leaflet was able to address salient knowledge gaps in the participants and remained sustainable over a two-week follow-up. Thus, its inclusion into future antibiotic awareness campaigns should be encouraged.

## Introduction

Over the decades, escalation of antimicrobial resistance has resulted in it becoming a global public health concern. The World Health Organization (WHO) defines antimicrobial resistance as the ability of a microorganism to resist an antimicrobial drug that was initially effective against it [1]. Misuse and overuse of antibiotics has resulted from multiple factors at the level of patients, healthcare providers, and healthcare systems, which has led to an acceleration of the occurrence of antibiotic resistance [2].

Consequently, common infectious diseases have resulted in greater morbidity, prolonged treatment, increased cost, and eventually more death [3]. Recently, resistance to colistin (polymyxin E), the ultimate antibiotic choice for life-threatening carbapenem-resistant Enterobacterales infections was found, which led to treatment failure [1]. With the antibiotic pipeline running dry, physicians are being left with limited therapeutic options.

The Malaysia National Medical Care Survey 2010 reported that upper respiratory tract infection (URTI) is the most commonly presenting illness in Malaysia, making up more than a quarter of primary care encounters, resulting in a consultation rate of 29.6% [4]. Half of the antibiotics prescribed in primary care were employed to treat URTI [5, 6]. Misuse of antibiotics for URTI has frequently been reported overseas [7, 8]. Given that the majority of the URTIs are viral in origin and the prevalence of streptococcal infections has not exceeded 20%, evidence of inappropriate antibiotic use is apparent [5, 6].

In view of a decline in the development of novel antibiotics, prudent use of available antibiotics plays a crucial role in curbing the emergence of antibiotic resistance [9]. According to Goossens H et al., a decline of outpatient antibiotic use by 36% between 1997 and 2006 has been associated with a substantial decrease in penicillin-, tetracycline-, and macrolide-resistant *Streptococcus pneumoniae* [10]. Concurrently, there was a significant decrease in macrolide resistance, from 17 to 2%, in *Streptococcus pyogenes* between 2001 and 2007 [10].

Previous studies reported that the public take antibiotics to help them recover faster, expect antibiotics to be prescribed for common cold, stop antibiotics when they start to feel better, share antibiotics with sick family members, and use left-over antibiotics for treating future respiratory illnesses [11, 12]. Lim and Teh [13] described that Malaysians have inadequate knowledge of antimicrobials, which was associated with more frequent and incorrect use of them. In Putrajaya, it was found out that more than 80% of the respondents failed to correctly identify that antibiotics do not eradicate viral infections [13]. As some physicians might be pressured to prescribe antibiotics for viral URTIs to meet patients’ expectations, public awareness on the appropriate use of antibiotic is important to reduce misuse.

Numerous studies proved that educational intervention can effectively improve knowledge and attitude toward medicines, including antibiotics [14–16]. The WHO also issued the ‘Global Strategy for Containment of Antimicrobial Resistance’ in 2001, urging member countries to initiate awareness and educational campaigns for patients and the general public on the appropriate use of antibiotics to combat antibiotic resistance [17].

Nevertheless, previous educational materials contained much information that was not targeted due to the gap in public knowledge [18, 19]. A targeted education tool with focused content is known to be an effective and feasible strategy when compared to generic messages in improving the public’s antibiotic knowledge [20, 21]. In addition, educational tools that include animation and pictures may be more efficient than those containing only plain text [20]. In light of the lack of a local intervention study, this research sought to determine the impact of targeted educational intervention on public knowledge and perception of antibiotic use and resistance in the state of Perak, Malaysia.

## Methods

This was an experimental study and data were collected via validated self-administered questionnaires. The respondents were required to answer the same set of questionnaires before the educational intervention, immediately post-intervention, and two-weeks post-intervention. The last questionnaire was conducted using telephone interviews. This study was registered in the Malaysia National Medical Research Registry (NMRR-18–2029-43,008) and obtained the approval of the Medical Research and Ethics Committee (MREC).

Adults who were over 18 years old and able to comprehend either the Malay or English language were included. Healthcare professionals (pharmacists, dentists, and physicians) were excluded from this study. Data collection was conducted in 20 public health clinics and five public tertiary hospitals in the Perak state of Malaysia from January to February 2019. One trained pharmacist was assigned from each study site to collect data and conduct the educational intervention. The respondents were selected via sequential sampling, where every 5^th^ subject who presented at the registration counter was recruited. Written informed consent was obtained from all the participants before their participation.

Using PS: Power and Sample Size Calculation (Version 3.0, January 2009), paired *t*-tests [22] to detect a 6.5-point increase in mean knowledge score with a significance level of 5%, a power level of 95%, and an SD of 19.14 based on a previous study taking into account a 10% non-participation rate and a 50% loss to follow-up rate, 288 respondents were required [21, 23].

The educational intervention tool was developed after reviewing the knowledge gaps concluded from previous studies [9, 24, 25]. The educational tool was a double-sided A4 leaflet containing brief, easily-understandable information on proper antibiotic use, causes of antibiotic resistance, and the responsibilities of the public to stem resistance. The materials were adapted from the Centres for Disease Control and Prevention, USA; the Ministry of Health, Malaysia; and the WHO [18, 19, 26, 27]. The educational intervention tool was initially developed in the English language, and subsequently translated into the Malay and Chinese language by the researchers. The content of the educational tool was reviewed by two senior infectious disease specialists and pre-tested among ten members of the public. It was then simplified and modified accordingly, so that it could at least be comprehended by a high school-level student (grade 8 level) before being finalised for use.

The questionnaire was initially developed in the English version, and subsequently translated into the Malay language using a paid, professional translation service. The questions were adapted from previous studies [13–15, 24, 25] and validated before use. Face and content validation of the questionnaire was performed by a senior academic and a senior clinical pharmacist in infectious disease management. Reliability testing was performed, and all the domains achieved a Cronbach’s alpha value of more than 0.7 during the pilot test. The respondents were allowed to choose whether they answered the Malay or the English version, as these two languages are the most widely spoken in the country. Chinese was offered only in the educational tool for respondents to understand the material in the language with which they were most comfortable. The researchers who performed the face-to-face and telephone interviews were trained over a four-hour session by the primary investigators to standardise the method of questionnaire administration in order to reduce subjectivity.

Each correct answer was given a score of 1, while no score was given to any incorrect answer. The questionnaire contained five sections: (i) the demographic data of respondents; (ii) experience in antibiotic use; (iii) knowledge of antibiotic resistance; (four items, maximum score: 4); (iv) knowledge of antibiotic use (six items, maximum score: 6); and (v) perception of antibiotic use (five items; maximum score: 5).

Collected data were entered using Microsoft Excel and analysed using IBM’s SPSS Statistics, version 21.0. Only participants who completed all three phases of the study [questionnaire before the educational intervention (Pre), immediately post-intervention (P1) and two-weeks post-intervention (P2)] were included in the final analysis. Respondents’ demographic characteristics and background experience were reported in a descriptive manner. A chi-square test was used to explore the association between demographic characteristics and the level of knowledge. Multiple linear regression analysis was performed to identify significant predictors of the total knowledge score. McNemar’s test was performed to analyse the difference between Pre and P1, P1 and P2, and Pre and P2. Repeated measures ANOVA was employed to measure changes in re-intervention and post-intervention mean scores.

## Results

There were 300 respondents recruited and 234 respondents (78%) who completed the study were included in the final analysis. Among them, a majority were Malay (162/234, 69.2%), female (143/234, 61.1%) and university graduates (94/234, 40.2%). The mean age was 40.7 ± 14.6 years. A quarter of respondents (59/234, 25.2%) declared having previous exposure to an antibiotic awareness campaign while half of the respondents (120/234, 51.3%) reported having a family member working as a healthcare professional. A total of 213 respondents (91.5%) claimed that they had taken antibiotics at least once in their lifetime with 46% of them (98/213) reported to have taken antibiotics less than once a year and 20 respondents (8.5%) claimed to be antibiotic-naïve (Table 1).

**Table 1:** Demographic characteristics of respondents

Characteristics (N = 234)	Frequency	%
Gender		
Male	91	38.9
Female	143	61.1
Age^#^	40.66 ± 14.56	
Ethnicity		
Malay	162	69.2
Chinese	38	16.3
Indian	29	12.4
Others	5	2.1
Academic qualification		
University	94	40.2
Secondary high school	81	34.6
Secondary lower school	45	19.2
Primary or lower	14	6.0
Exposure to antibiotic awareness campaign		
Yes	59	25.2
No	175	74.8
1st degree family member in health care related job		
Yes	120	51.3
No	114	48.7
Duration of Last Taken Antibiotic (N = 233*)		
Within one month ago	24	10.3
Within 6 months	59	25.3
Within 1 year	31	13.3
More than 1 year ago	99	42.5
Have not taken before	20	8.6
Frequency of Antibiotic Consumption (N = 233*)		
At least once a month	3	1.3
At least once every 3 months	12	5.1
At least once every 6 months	36	15.4
At least once a year	64	27.5
Less than once a year	98	42.1
Have not taken before	20	8.6
Source of Antibiotic (N = 213)**		
Own (leftover/sharing)	4	1.9
Prescribed by doctor	205	96.2
Purchase at retail pharmacy with prescription	6	2.8
Purchase at retail pharmacy without prescription	9	4.2
Private clinic without seeing doctor	5	2.3

^#^Expressed as mean ± standard deviation

*One missing data

**Multiple selection was allowed

**Respondents whom had never taken antibiotic before were excluded

Out of the 213 respondents who had been exposed to at least one antibiotic in their lifetime, 18 admitted that they had initiated an antibiotic treatment on their own without consulting a doctor. Those with previous exposure to antibiotic campaigns have a lower tendency to self-medicate (2/57, 3.5%) when compared to those without prior exposure to the campaigns (22/156, 14.1%; *p* = 0.030).

### Knowledge of antibiotics resistance

The knowledge level of antibiotic resistance was generally high. The number of respondents that answered statement KR2 correctly increased by 26.1% after the educational intervention (Pre: 150/234, 64.1%; P2: 211/234, 90.2%; *p* < 0.001). Correct responses to statement KR4 (Pre: 163/234, 69.7%) also increased significantly at the two-weeks post-intervention assessment (208/234, 88.9%; *p* < 0.001) (Table 2).

**Table 2:** Knowledge on antibiotics resistance

Code	Statements	Pre-intervention [Pre] (N = 234)	Post-intervention		*p *Value_*_
			Immediate [P1] (N = 234)	2 weeks [P2] (N = 234)	
		Correct responses n (%)	Correct responses n (%)		
KR1	Antibiotic resistance happens when the antibiotic loses its ability to treat bacterial infections	160 (68.4)	221 (94.4)	212 (90.6)	< 0.001
KR2	Taking leftover antibiotics from previous infection may cause antibiotic resistance	150 (64.1)	213 (91.0)	211 (90.2)	< 0.001
KR3	Treatment may fail if you do not finish antibiotics as instructed by doctors	187 (79.9)	227 (97.0)	227 (97.0)	< 0.001
KR4	Using antibiotics without consulting doctor or purchasing directly from the pharmacy without a prescription may cause antibiotic resistance	163 (69.7)	219 (93.6)	208 (88.9)	< 0.001

*Mc Nemar test was performed to analyse the difference between Pre vs P1, P1 vs P2 and Pre vs P2. The *p *values for Pre-intervention vs Post-intervention (immediate) and Pre-intervention vs Post-2-weeks-intervention were presented in the table (*p* < 0.001). *p *values for Post-intervention (immediate) vs Post-2-weeks-intervention were insignificant for all questions (*p* > 0.05)

### Knowledge on antibiotics use

Before the intervention, only a quarter of respondents (60/234, 25.6%) understood that antibiotics could not treat viral infections. Significant improvement was observed after the educational intervention (P2: 188/234, 80.3%; *p* = 0.004). Notably, only 9.8% (Pre: 23/234) respondents were able to identify the types of conditions that can be treated using antibiotics. There was a significant improvement after the educational intervention (P2: 101/234, 43.2%; *p* = 0.022) (Table 3).

**Table 3:** Knowledge on Antibiotics Use

Code	Statements	Pre-intervention [Pre] (N = 234)	Post-intervention		*p *Value _*_
			Immediate [P1] (N = 234)	2 weeks [P2] (N = 234)	
		Correct responses n (%)	Correct responses n (%)		
KU1	Antibiotics can treat the following condition(s): Fever and sore throat for a day; Fever with running nose; Inflammation/Swelling; Common cough and wheezing; Influenza; None of the above	23 (9.8)	118 (50.4)	101 (43.2)	< 0.001
KU2	Viral infections can be treated by taking antibiotics	60 (25.6)	206 (88.0)	188 (80.3)	< 0.001
KU3	Taking antibiotics early can prevent myself from infection/illness	106 (45.3)	195 (83.3)	194 (82.9)	< 0.001
KU4	Taking antibiotics for common cough and wheezing can help me to recover faster as compared to not taking any	90 (38.5)	172 (73.5)	171 (73.1)	< 0.001
KU5	Taking a low dose of antibiotic is better than not taking any dose	85 (36.3)	170 (72.6)	166 (70.9)	< 0.001
KU6	Antibiotic resistant bacteria can be spread from one person to another	112 (47.8)	201 (85.9)	190 (81.2)	< 0.001

*Mc Nemar test was performed to analyse the difference between Pre vs P1, P1 vs P2 and Pre vs P2. The *p *values for Pre-intervention vs Post-intervention (immediate) and Pre-intervention vs Post-2-weeks-intervention were presented in the table (*p* < 0.001). *p *values for Post-intervention (immediate) vs Post-2-weeks-intervention were insignificant for all questions (*p* > 0.05) except for question ‘antibiotic can treat the following condition’ (*p* = 0.022) and ‘viral infection can be treated by antibiotic’ (*p* = 0.004) where the reduction in correct responses between Post-intervention (immediate) and Post 2-weeks-intervention is significant

### Perception of antibiotic use

Only a minority of the respondents (62/234, 26.5%) believed that they had a role to play in stopping antibiotic resistance. A significant improvement (162/234, 69.2%) was observed at two-weeks post-intervention (*p* < 0.001). There was also an increase in concern about the impact of antibiotic resistance on their health and family (Pre: 155/234, 66.2%; P2:203/234, 86.8%; *p* < 0.001). A significant improvement in respondents’ perception on antibiotic use was observed after the educational intervention (*p* < 0.001) (Table 4).

**Table 4:** Perception on Antibiotics Use

Code	Statements	Pre-intervention [Pre] (N = 234)	Post-intervention		*p *Value_*_
			Immediate [P1] (N = 234)	2 weeks [P2] (N = 234)	
		Correct responses n (%)	Correct responses n (%)		
PU1	Individual who take antibiotics should stop their treatment earlier than recommended when symptoms improve	157 (67.1)	214 (91.5)	212 (90.6)	< 0.001
PU2	Doctors shall always prescribe antibiotics if someone is having fever, sore throat or runny nose	101 (43.2)	196 (83.8)	191 (81.6)	< 0.001
PU3	Antibiotics should be made available without a doctor’s prescription	188 (80.3)	219 (93.6)	220 (94.0)	< 0.001
PU4	I am not worried about the impact of antibiotic resistance on my health and my family	155 (66.2)	204 (87.2)	203 (86.8)	< 0.001
PU5	There is not much that I can do to stop antibiotic resistance	62 (26.5)	156 (66.7)	162 (69.2)	< 0.001

*Mc Nemar test was performed to analyse the difference between Pre vs P1, P1 vs P2 and Pre vs P2. The *p *Values for Pre-intervention vs Post-intervention (immediate) and Pre-intervention vs Post-2-weeks-intervention were presented in the table (*p* < 0.001). *p *Values for Post-intervention (immediate) vs Post-2-weeks-intervention were insignificant for all questions (*p* > 0.05)

### Regression model

A multiple linear regression was performed to predict the total mean score two weeks after the intervention by controlling for age, gender, educational level, ethnicity, exposure to previous campaigns, self-medication history, and professional status of relatives. The regression model was as follows:

$${\text{Total}}\;{\text{score}}\;{\text{after}}\;{\text{two}}\;{\text{weeks}} = {1}0.{633} - {1}.{149}\left( {{\text{Secondary}}\;{\text{lower}}\;{\text{education}}} \right) - {1}.{81}0 \, \left( {{\text{primary}}\;{\text{education}}} \right) - {1}.{562}\left( {{\text{Indian}}\;{\text{ethnicity}}} \right) + 0.{921}\left( {{\text{Female}}} \right) + {1}.{483}\left( {{\text{self}} - {\text{medication}}} \right) \, \left[ {{\text{F}}\left( {{11},{221}} \right) = {4}.0{5},\;p < 0.00{1},\;R^{{2}} = 0.{168}} \right]$$

### Mean knowledge and perception score

The mean knowledge score on antibiotic use significantly increased from 2.03 ± 1.56 (Pre) to 4.32 ± 1.48 (P2). Similarly, the mean knowledge score on antibiotic resistance increased from 2.83  ± 1.28 (Pre) to 3.67  ± 0.78 (P2) while the mean knowledge score on antibiotic perception also improved from 2.83  ± 1.38 (Pre) to 4.22 ± 1.02 (P2). All increments were significant (Pre-P2: *p* < 0.001).

The mean overall score in the pre-intervention, immediate post-intervention, and two-weeks post-intervention assessment were 7.68 ± 3.31, 12.57 ± 2.50, 12.21 ± 2.57, respectively. The overall score improved significantly after the intervention, and no significant drop was observed two-weeks post-intervention (Pre-P1: *p* < 0.001; Pre-P2: *p* < 0.001; P1-P2: *p* = 0.072). (Table 5).

**Table 5:** Mean knowledge and perception score

Domains	Pre-intervention [Pre]	Post-intervention		Phase differences *p *value**
		Immediate [P1]	2 weeks [P2]	
	Mean (SD)	Mean (SD)		
Antibiotics resistance knowledge score	2.83 (1.28)	3.76 (0.62)	3.67 (0.78)	< 0.001_*_^^^
Antibiotics use knowledge score	2.03 (1.56)	4.56 (1.46)	4.32 (1.48)	< 0.001_*_^#^
Antibiotics perception score	2.83 (1.38)	4.25 (1.06)	4.22 (1.02)	< 0.001_*_^^^
Total score	7.68 (3.31)	12.57 (2.50)	12.21 (2.57)	< 0.001_*_^^^

*Significant differences were found between pre: p1, pre: p2 ^#^p1: p2 (*p* = 0.013) ^^^p1:p2 (*p* > 0.05)

**p Value generated by repeated measure ANOVA (Wilk’ lambda; Bonferroni Adjustment were made for multiple comparisons)

### Demographic characteristics associated with level of knowledge

#### Prior exposure to an antibiotic awareness campaign

At the beginning, respondents who were exposed to a previous antibiotic awareness campaign scored higher in all three domains in comparison to those without prior exposure (total score: 9.28 ± 3.12 vs 7.15 ± 3.21; *p* < 0.001; antibiotic use mean score: 2.60 ± 1.59 vs 1.84 ± 1.5; *p* = 0.001; antibiotic resistance mean score: 3.22 ± 1.07 vs 2.70 ± 1.32 *p* = 0.003; antibiotic perception mean score: 3.48 ± 1.25 vs 2.62 ± 1.36 *p* < 0.001). However, the score difference between the two groups was not significant at the two-weeks post-intervention assessment for all domains.

#### A healthcare professional as a family member

Prior to intervention, those respondents with family members working as healthcare professionals had a significantly higher total score (8.27 ± 3.28, *p* = 0.006), antibiotic use mean score (2.25 ± 1.55, *p* = 0.031), and antibiotic perception mean score (3.12 ± 1.28 *p* = 0.001). At two-weeks post-intervention, the mean score between the two groups did not differ statistically.

## Discussion

This study reveals the inadequate knowledge and inappropriate perception on antibiotic use among the public in the state of Perak, Malaysia. While respondents’ knowledge of antibiotics resistance was satisfactory, most remained ignorant to the types of infection and illnesses which antibiotics can treat. Respondent’s knowledge and perception improved significantly post-intervention and was sustained after two weeks. The employment of focused one-on-one educational intervention may provide additional benefits in addition to a mass awareness campaign. Consistent with local and foreign studies, targeted educational interventions have shown great effectiveness in improving public knowledge, and perception on antibiotic use and resistance [15, 28–30].

Pre-intervention, 36% of the respondents were not aware that self-medication behaviour, such as taking leftover antibiotics or purchasing antibiotics without doctor’s consultation, accelerates the process of antibiotic resistance. The post-intervention evaluation showed significant improvement, in which 60% of the respondents acknowledged that the use of antibiotics without professional clinical judgments may contribute to antibiotic resistance. Comparatively, a previous study in Jordan with a similar intervention reported a less pronounced improvement (11%) [21] compared to this study, in which the sociodemographic information of the respondents was not noted. Hence, we could not confirm whether level of education or other sociodemographic characteristics contributed to this difference in the effectiveness of the intervention between the two studies.

In terms of knowledge on antibiotic use, the majority of the respondents were not able to identify illnesses treatable by antibiotics [31–34]. Similar public awareness surveys reported that 61% and 75% of respondents from China and India, respectively, believed antibiotics could treat illnesses of a viral origin, such as a cold and the flu [33].

In a recent local study, 52.7% respondents agreed that antibiotics could work on viruses [24]. To address this, we incorporated antibiotic indication and taglines, such as ‘Viruses and Bacteria: What’s got you sick?’ into our education material. At baseline, one quarter of the respondents (25.6%) in this study were aware that antibiotics were not effective against viruses. A significant improvement was observed post-intervention, where more than 80% of the respondents answered correctly. The reduction of antibiotic demand for a typical viral infection in the European Antibiotic Awareness Day campaign evaluation suggested continuous education regarding infections of viral and bacterial origin should be prioritised in future antibiotic awareness campaigns [31].

While knowledge of antibiotics was significantly associated with prudent antibiotic use, public perception was equally important. Three-quarters of the respondents perceived that they did not have a role in curbing antibiotic resistance before the intervention. Respondents were worried about the consequences of antibiotic resistance (KR1&PU4) but were not convinced that they could help in the fight against it. These findings were similarly reported in a previous survey, where around three-quarters of respondents in Vietnam, Indonesia, and China agreed that there was little they could do in the antibiotic resistance fight [33]. Our intervention significantly enhanced the individual awareness in responsibility for combating antibiotic resistance, hence this message should be emphasised in future awareness campaigns.

Furthermore, more than half of the respondents had inappropriate expectations for antibiotics when having fever, sore throat, or runny nose. Similar responses were observed in Poland, where 41% of respondents expected antibiotics for flu while 23% expected antibiotics when having sore throat [31]. Misaligned beliefs and demands for antibiotics by patients may exert pressure on healthcare providers, hence leading to inappropriate prescription to meet the patients’ satisfaction [35, 36]. The educational material in this study contained several key facts on the ineffectiveness of antibiotics against viral infections and significant improvement was observed after the intervention. Incorporation of key facts, such as ‘Cold? Flu? Take care without antibiotics! Antibiotics don’t kill viruses and don’t treat viral illnesses’ in previous awareness campaigns demonstrated effectiveness in reducing the expectations of antibiotics in treating viral respiratory infections [31].

It is important to note that respondents with at least one family member working as a healthcare professional possessed better antibiotic knowledge and perception at baseline. Public preference to seek informal advice from family and friends rather than formal healthcare was observed in previous studies [37, 38]. These observations were in-line with previous recommendations and highlighted social network support as one of the measures in promoting public health [39, 40]. The importance of community influence was also crucial, as demonstrated by the introduction of the Antibiotic Guardian campaign in the United Kingdom, which effectively reduced antibiotic resistance and encouraged sharing of knowledge [41–43]. In Malaysia, the ‘Know Your Medicine Ambassador’ programme, showed an 81.4% increase in awareness on quality use of medications [44]. Therefore, integrating antibiotic-related information into the ‘Know Your Medicine’ programme may be beneficial in promoting prudent antibiotics use.

This study has several strengths. Our intervention tool (an educational leaflet) was designed to address several knowledge gaps among the Perak state population, based on a previous study by Choo et al. [24]. Unlike previous educational leaflets, our focus was to target the misconception of antibiotic effectiveness on upper respiratory infection, especially, and the public’s ability to identify viral infections in which respondents were found to be lacking [24]. Instead of conventional modalities via a lecture-style distribution of generic information, we adopted a more focused, individualised, and interactive counselling approach that increased the effectiveness of the message delivered [45, 46]. We also developed the leaflets in the three major languages: Malay, English, and Chinese, in order to overcome the language barrier which is a common challenge in a multiracial country like Malaysia.

This study had its limitation: the findings only reflected the population in Perak (i.e. the state level) and not the entire country. We calculated the sample size based on previous literature^21^, which assumed a substantial increase in the mean knowledge score. This resulted in a smaller sample size, which might have affected the power of our study. Due to the fact that the questionnaires were available in multiple languages and majority of the respondents had a higher level of education, the intervention’s impact might have been skewed towards a more effective direction. While previous literature found that better knowledge was associated with more prudent antibiotic use^24^, we did not assess the participants’ practice of using of antibiotics; hence, we could not affirm this relationship. Non-response bias may present, as those who did not respond to the questionnaire may differ in terms of attitude towards antibiotics and willingness to acquire new information, compared to those who responded.

The impact of face-to-face interactive and targeted educational intervention was proven in this study. In the future, expanding the coverage of the antibiotic awareness campaign through a digital or online interactive web-based medium may be useful to enhance public awareness—further studies are certainly warranted.

## Conclusion

The face-to-face, targeted educational intervention method effectively improved respondents’ antibiotic resistance knowledge and antibiotic use knowledge and perception. This understanding was also sustained over two weeks. The implementation of educational material tailored according to the audience’s knowledge gap is warranted in future antibiotic awareness campaigns.

## Data Availability

All data generated or analysed during the current study are available from the corresponding author on reasonable request.
